# 4-(1*H*-Pyrazol-1-yl) Benzenesulfonamide Derivatives: Identifying New Active Antileishmanial Structures for Use against a Neglected Disease

**DOI:** 10.3390/molecules171112961

**Published:** 2012-11-01

**Authors:** Roberta K. F. Marra, Alice M. R. Bernardino, Tathiane A. Proux, Karen S. Charret, Marie-Luce F. Lira, Helena C. Castro, Alessandra M. T. Souza, Cesar D. Oliveira, Júlio C. Borges, Carlos R. Rodrigues, Marilene M. Canto-Cavalheiro, Leonor L. Leon, Veronica F. Amaral

**Affiliations:** 1 Programa de Pós-graduação em Química, Instituto de Química, Universidade Federal Fluminense, Outeiro de São João Baptista, Niterói, RJ, 24020-150, Brazil; Email: katlenfusco@bol.com.br (R.K.F.M.); posgraduacaouff@vm.uff.br (C.D.O.); juliusborges@yahoo.com.br (J.C.B.); 2 Programa de Pós-graduação em Ciências e Biotecnologia PPBI, Instituto de Biologia, Universidade Federal Fluminense, Outeiro de São João Baptista, Niterói, RJ, 24020-150, Brazil; Email: tathimir@hotmail.com (T.A.P.); marie.biouff@gmail.com (M.-L.F.L.); 3 Laboratório de Bioquímica de Tripanosomatídeos, Instituto Oswaldo Cruz, Fundação Oswaldo Cruz, RJ, 21040-900, Brazil; Email: karenbio@hotmail.com (K.S.C.); mcantocavalheiro@hotmail.com (M.M.C.-C.); lleon@ioc.fiocruz.br (L.L.L.); 4 Faculdade de Farmácia, ModMolQSAR, Universidade Federal do Rio de Janeiro, Rio de Janeiro, RJ, 21941-590, Brazil; Email: amtsouza2@yahoo.com.br (A.M.T.S.); rangelrodrigues2003@yahoo.com.br (C.R.R.)

**Keywords:** pyrazoles, benzenesulfonamide, *Leishmania*, cytotoxicity, *in silico* evaluation

## Abstract

Leishmaniasis is a neglected disease responsible for about 56,000 deaths every year. Despite its importance, there are no effective, safe and proper treatments for leishmaniasis due to strain resistance and/or drug side-effects. In this work we report the synthesis, molecular modeling, cytotoxicity and the antileishmanial profile of a series of 4-(1*H*-pyrazol-1-yl)benzenesulfonamides. Our experimental data showed an active profile for some compounds against *Leishmania infantum* and *Leishmania amazonensis*. The profile of two compounds against *L. infantum* was similar to that of pentamidine, but with lower cytotoxicity. Molecular modeling evaluation indicated that changes in electronic regions, orientation as well as lipophilicity of the derivatives were areas to improve the interaction with the parasitic target. Overall the compounds represent feasible prototypes for designing new molecules against *L. infantum* and *L. amazonensis*.

## 1. Introduction

Leishmaniasis is a parasitic disease with severe morbidity and mortality rates. According to the World Health Organization, there are two million infected people and approximately 56,000 deaths reported every year [1,2]. Ninety percent of the visceral leishmaniasis (VL) cases occur in Brazil, India, Nepal, and Bangladesh [1,2]. Thus leishmaniasis is included in the WHO neglected diseases group since the pharmaceutical industry has little commercial incentive to invest in new treatment options.

*Leishmania* spp. causes a broad spectrum of infectious diseases ranging from self-healing cutaneous ulcerations to progressive and lethal visceral infection. *L. amazonensis* infection in humans can be associated with a spectrum of disease manifestations, depending on the immune status of the host or other external factors [3]. Currently the epidemiological pattern of *Leishmania* spp. [e.g., *L. amazonensis* and *L. infantum* (*L. chagasi* syn.)] is changing, with a tendency to urbanization and geographic expansion. Despite the high worldwide prevalence, few advances were made in the treatment of this disease [4,5,6,7,8,9]. There are no vaccines for Leishmaniasis and vector control is complex [2,8].

Currently, the drugs used for leishmaniasis treatment present many disadvantages, including serious clinical side effects such as nephrotoxicity, hepatotoxicity and cardiac arrhythmia, whereas the emerging strain resistance to available drugs has also decreased the treatment options [7]. Resistance to pentavalent antimonials is generating a problem in the treatment of visceral leishmaniasis in India whereby naturally resistant parasites have higher virulence than susceptible *L. donovani* [9]. Some additional drugs including pentamidine (aromatic diamine), amphotericin B (polyene antibiotic) and miltefosine (alkyl phopholipid) were introduced as substitutes in chemotherapy but without complete efficacy [4]. Currently the pentavalent antimonials are the first line treatment employed in Brazil, followed by pentamidine and amphotericin B [10].

The leishmaniasis treatment restrictions and the resistant strains point to the urgent need for new therapeutic options. Therefore, several reports regarding natural and synthetic new antileishmanial compounds have been described [4,7], including some by our group [11,12,13]. Literature about the pyrazole nucleus chemistry has reported a broad spectrum of pharmaceutical activities [14]. Similarly, sulfonamides show different biological activity profiles, including antibacterial [15], anti-HIV [16], anti-*Trypanosome* [17,18] and anti- *Leishmania* properties [17,18,19,20].

Recently, we reported new pyrazole carbohydrazide derivatives with *in vitro* antiparasitic activity against *L. amazonensis* promastigotes and, to a lesser extent, *L. braziliensis* and *L. infantum (chagasi syn.)*, with no toxicity to murine macrophages [11]. The literature also described that mice experimentally infected with *L. amazonensis* and treated with pyrazole carbohydrazide derivatives controlled the evolution of both footpads cutaneous lesions and dissemination of parasites to draining lymph nodes [6]. In addition the evaluation of pyrazole carboximidamide derivatives also revealed potential *in vitro* activity against *L. amazonensis* [12].

A therapy using an anti-inflammatory drug with antileishmanial properties, lower toxicity, cost, side effects and patient compliance may be very advantageous [6]. Previous reports from our laboratory described the *in vitro* and *in vivo* activity of pyrazoles against *Leishmania* parasites [6,11,12,13]. In this work we describe the synthesis of a new pyrazole family exploring this time the addition of a sulfonamide group. Thus we evaluated the activity of these 4-(1*H*-pyrazol-1-yl)benzenesulfonamide derivatives against *Leishmania infantum* and *L. amazonensis* and their cytotoxicity profile towards mammalian cells. We also performed a structure-activity relationship (SAR) evaluation of these derivatives using a molecular modeling approach. 

## 2. Results and Discussion

### 2.1. Chemistry

This work examined the functionalization of the core 1-phenylpyrazoles with a sulfonamide due to the group’s antileishmanial profile previously described in the literature [11]. The 4-(4-bromo-5-chloro-3-methyl-1*H*-pyrazol-1-yl)benzenesulfonyl chloride (**1**) derivative was obtained in good yield by a regioselective electrophilic aromatic substitution reaction between the corresponding 1-phenylpyrazole derivative and chlorosulfonic acid. The compound 4-(5-chloro-3-methyl-1*H*-pyrazol-1-yl)phenylamine (**2**) was prepared by nitration of the corresponding 1-phenylpyrazole derivative, followed by reduction with iron powder and ammonium chloride. 

**Scheme 1:**
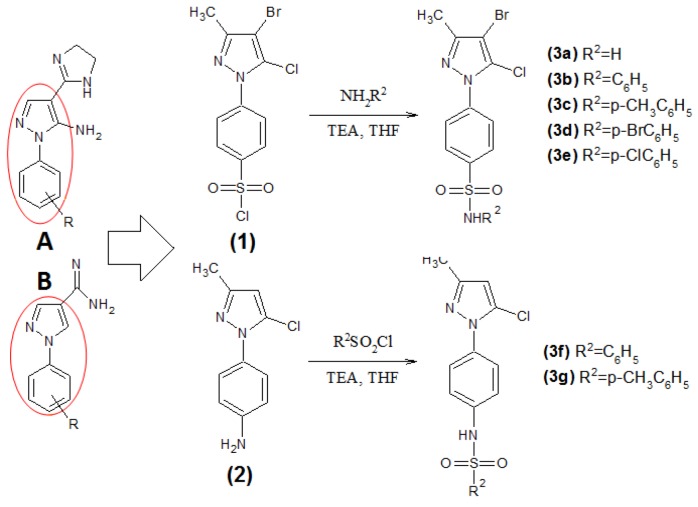
Synthesis of 4-(1*H*-pyrazol-1-yl)benzenesulfonamide derivatives **3a**–**g** based on the previous synthesis of 5-amino-1-aryl-4-(4,5-dihydro-1*H*-imidazol-2-yl)-1*H*-pyrazoles (**A**) and 1-aryl-1*H*-pyrazole-4-carboximidamides (**B**).

The target compounds **3a**–**g** could be easily prepared with these key intermediates in hand through substitution reactions between the sulfonyl chloride moiety and amino intermediates [11,17]. Sulfonyl chlorides are electrophilic reagents that react readily with primary and secondary amines, such as the NH_2_ of the benzene ring as shown in Scheme 1.

These reactions were rapid with no side products, according to the experimental data. All the synthesized compounds were obtained as solids, purified by recrystallization from ethanol and characterized by spectroscopic techniques (IR, ^1^H-NMR and ^13^C-NMR) and elemental analysis. The IR spectra of the compounds showed the presence of the characteristic bands for SO_2_ in the 1330–1630 cm^−1^ range. In addition, the ^1^H-NMR spectra of these compounds revealed the presence of a singlet amino (NH) peak and multiplets due to the aromatic protons.

### 2.2. Biological Evaluation

In this work we evaluated the biological effect of 4-(1*H*-pyrazol-1-yl)benzenesulfonamides derivatives **3a**–**g** against both the *L. infantum* (*L. chagasi* syn.) and *L. amazonensis* promastigote forms and compared with them with pentamidine, a reference drug used in leishmaniasis treatment [4] similar to other reports in the literature [21,22,23]. Interestingly, **3b** and **3e** showed the best *in vitro* active profile against the infective *L. amazonensis* promastigotes forms (IC_50_ = 0.070 mM and 0.072 mM, respectively) as well as against *L. infantum* (*L. chagasi* syn.) (IC_50_ = 0.059 mM and 0.065 mM, respectively) as shown in Table 1.

**Table 1:** Comparison of the antileishmanial (IC_50_) effect against *Leishmania* spp. and the theoretical parameters evaluation of the molecular electronic properties of the new 4-(1*H*-pyrazol-1-yl)benzenesulfonamide series **3a**–**e**, including the lowest unoccupied molecular orbital (LUMO) energy (eV), dipole (Debye), and Lipinski “rule of five” (molecular weight - Mw, number of hydrogen bound donor - HBD, or acceptor - HBA groups and lipophilicity - cLogP).

**Compound**	**Antileishmanial activity **				**LUMO** (Ev)	**Dipole** (Debye)	**Lipinski “rule of five”**			
	(IC_50_ = mM) ^a,b^									
	*Leishmania infantum*	S.I ^c^	*Leishmania amazonensis*	S.I ^c^			M_W_	clogP	HBA	HBD
**3a**	0.228 ± 0.19	0.78	0.228 ± 0.33	0.78	−1.61	4.61				
**3b**	**0.059 ± 0.01**	**2.44**	**0.070 ± 0.02**	**2.05**	**−1.67**	**4.53**	**426.72**	**3.27**	**4**	**1**
**3c**	0.123 ± 0.05	1.33	0.318 ± 0.59	0.51	−1.63	4.80	440.75	3.58	4	1
**3d**	0.099 ± 0.08	0.49	0.075 ± 0.01	0.65	−1.79	3.69	505.62	3.97	4	1
**3e**	**0.065 ± 0.04**	**1.78**	**0.072 ± 0.05**	**1.61**	**−1.77**	**3.75**	**461.17**	**3.88**	**4**	**1**
**3f**	0.138 ± 0.11	0.76	0.153 ± 0.22	0.68	−1.29	4.84	347.83	2.57	4	1
**3g**	0.149 ± 0.12	1.21	0.136 ± 0.054	1.33	−1.18	5.24	361.85	2.88	4	1

^a^ Mean of IC_50_ (mM) ± S.D. for three determination; ^b^ Pentamidine was used as control drug (IC_50_ = 0.062 and 0.021 mM; S.I = 0.87 and 2.57 respectively); ^c^ Selectivity index (SI): CC_50_ drug/IC_50_ drug.

*L. infantum (L. chagasi syn.)* is a strain of epidemiological importance [24] and responsible for the American visceral clinic form, whereas *L. amazonensis* has been implicated in cutaneous, mucosal, visceral and diffuse clinic forms of leishmaniasis [3]. Our biological data pointed to the potential of compounds **3b** and **3e** as active pyrazole structures containing sulfonamide groups for treating infections caused by these two *Leishmania* strains. 

Our antileishmanial results with this new series are in agreement to the previous literature that shows the sulfonamide functionality can display antiparasitic [17,18,19,20] as well as antibacterial [13,25] and anti-viral HIV activities [16]. In fact the addition of the sulfonamide together with the bromide improved the antileishmanial activity of this series compared to our previous data from 5-amino-1-aryl-4-(4,5-dihydro-1*H*-imidazol-2-yl)-1*H*-pyrazoles derivatives [12] (Scheme 1). The activity against *L. amazonensis* of our new pyrazole series leads **3b** and **3e**, was slightly better than that of 1-aryl-1*H*-pyrazole-4-carboximidamides derivatives (lowest IC_50_ = 0.105 mM) [12] but not than that of 5-amino-1-aryl-4-(4,5-dihydro-1*H*-imidazol-2-yl)-1*H*-pyrazoles (lowest IC_50_ = 0.015 mM). The antileishmanial profile against *L. infantum* was also evaluated for 5-amino-1-aryl-4-(4,5-dihydro-1*H*-imidazol-2-yl)-1*H*-pyrazoles) [13]. Our results reveal this new series is active and better (**3b** and **3e** IC_50_ = 0.059 mM and 0.065 mM, respectively) compared to the non-active profiles of the 1-aryl-4-(4,5-dihydro-1*H*-imidazol-2-yl)-1*H*-pyrazole derivatives (lowest IC_50_ > 0.500 mM).

**Figure 1:**
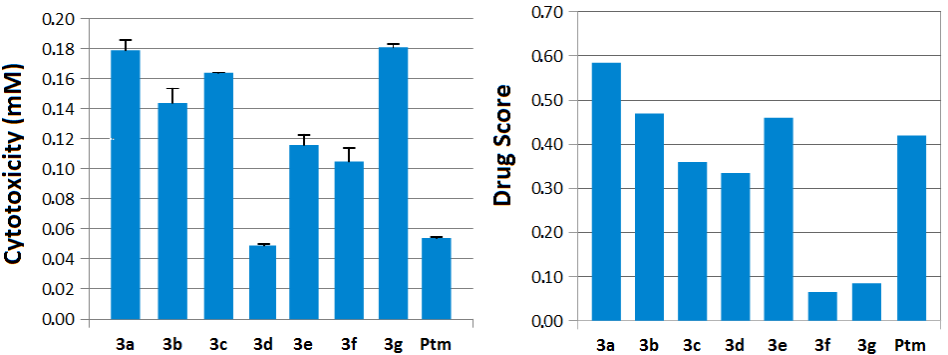
The experimental cytotoxicity (CC_50_) using murine adherent peritoneal cells and the theoretical drugscore values of the new 4-(1*H*-pyrazol-1-yl)benzenesulfonamide series (**3a**−**g**) compared with pentamidine (Ptm), a current antileishmanial drug on the market. Higher values on both analyses suggest a good drug profile.

Importantly, **3b** showed an antileishmanial activity against both *Leishmania* species (IC_50_ = 0.059 mM and 0.070 mM) and comparable to the effect of pentamidine on *L. infantum* (IC_50_ = 0.062 mM) (Table 1). These data suggest that this derivative has the most potential for activity against *L. infantum* strains as an alternative to pentamidine. This is also reinforced by the cytotoxic evaluation using murine peritoneal adherent cells that showed **3b** (CC_50_ = 0.144 mM) and **3e** (CC_50_ = 0.116 mM) with lower cytotoxicity than pentamidine (CC_50_ = 0.054 mM) (Figure 1). The selectivity index to *L. infantum* reinforced the fact that compound **3b** (2.44) as better than pentamidine (0.87). Overall the *in vitro* results pointed to further explore these molecules in the *in vivo* tests as described for this and for other synthetic derivatives [25,26,27,28].

### 2.3. Molecular Modeling Data

In order to identify structural features important to this series’ antileishmanial profile we performed a structure-activity relationship (SAR) evaluation using a molecular modeling approach. The derivatives 3D-structures were constructed using the Spartan 10 program and the molecular properties were calculated as described in the Supplementary Material. Several parameters were evaluated, including the highest energy occupied molecular orbital (HOMO) and lowest energy unoccupied molecular orbital (LUMO). They are known as frontier orbitals or interacting molecular orbitals and a pair that lies closest in energy of any pair of orbitals in two molecules that interact, which allows them to interact most strongly. Therefore, it can be detected a correlation between the biological activity HOMO and LUMO energy and/or distribution as they may be directly involved in the interaction with the target [29,30].

The overall analysis pointed the lowest LUMO energy and dipole moment as well as the highest theoretical lipophilicity (cLog P) of the most active compounds as structural features that may contribute to the biological activity in this series. These features are probably related to the ability of penetrating the biological membranes of the parasite and interact with the biological target (Table 1). 

**Figure 2:**
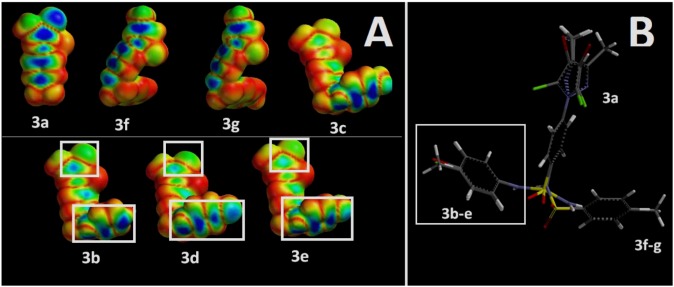
Theoretical structural analysis of the new 4-(1*H*-pyrazol-1-yl)benzenesulfonamide series (**3a**–**g**) using a molecular modeling approach. Comparison of the highest occupied molecular orbital (HOMO) distribution (**A**) and of the structural orientation through the superposition (**B**) of the less (compounds **3a**, **3f**, **3g** and **3c**) and most active (**3b**–**e**) compounds. The theoretical analysis allowed pointing derivatives electronic regions (**A**) and orientation (**B**) that are probably related with a better interaction with the parasitic target (white boxes).

According to our theoretical structural analysis the absence of the aromatic substituent affected the antileishmanial activity (*i.e.*, **3a**), in agreement to the literature [23]. The analysis of the minimum energy conformations of these compound showed that compounds **3b**−**e**, **3f** and **3g** are coplanar, but with different spatial orientation (Figure 2). This is probably due to the retroisosterism of the sulfonamide, where the sulphur atom is linked directly to the aromatic ring in derivatives **3a**–**e**, whereas for **3f** and **3g**, the nitrogen atom of the sulfonamide is connected to the ring (Figure 2). This variation led to different orientations that should influence the biological activity in this series as the spatial complementarity is a requirement to interact with the biological target (Figure 2).

The stereo-electronic features evaluation suggests that the HOMO energy level (not shown) and molecular weight have no direct correlation with the observed activity (Table 1). However compounds **3b**–**e** exhibit different HOMO distribution profiles, which probably orient the interactions with the parasitic target (Figure 2).

The analysis of the steric parameters pointed to the importance of the substitution on the aromatic ring for the antileishmanial activity. Apparently a bigger substituent such as bromine (e.g., **3d**) may cause some steric hindrance at the molecular interaction level and slight HOMO distribution profile differences resulting in a low antileishmanial profile. Meanwhile, smaller (e.g., chlorine) or no substituents on the aromatic ring may properly interact as in **3e** and **3b**, respectively (Table 1 and Figure 2).

The 4-(1*H*-pyrazol-1-yl)benzenesulfonamide derivatives **3a**–**g** were also submitted to an *in silico* pharmacokinetics properties evaluation. Since good absorption is necessary for oral administration, we analyzed these derivatives according to the rule-of-five developed by Lipinski co-workers (Table 2) [31]. The rule-of-five indicates the theoretical potential for a chemical compound to have good oral bioavailability. The rule states that the most “druglike” molecules present clogP ≤ 5, molecular weight (MW) ≤ 500, number of hydrogen bond acceptors ≤ 10 and donors ≤ 5. Molecules violating more than one of these rules may have bioavailability problems. Our results showed that all compounds of the 4-(1*H*-pyrazol-1-yl)benzenesulfonamide (**3a**–**g**) fulfilled the Lipinski “rule-of-five”. Importantly, according to the theoretical analysis of the lipophilicity (clog P), the most active inhibitors of *L. amazonensis* and *L. infantum* were sufficiently hydrophobic for penetrating the biological membranes. 

We also compared the drugscore values of these new derivatives with pentamidine, an antileishmanial drug currently in use in the market (Figure 1). The most active compounds showed drugscore values (**3b** and **3e** = 0.48 and 0.46, respectively) similar to pentamidine (0.45) (Figure 1), revealing their potential profile similar to drugs current on the market. Far from establishing absolute results, the molecular modeling data obtained in this work using this series may help to design new benzenesulfonamide-related compounds more activity and/or safety against leishmaniasis. 

## 3. Experimental

### 3.1. Chemistry

#### 3.1.1. General

The chemicals were obtained from commercial supplies and used without purification, unless otherwise noted. Reactions were routinely monitored by thin-layer chromatography (TLC) on silica gel (60 F-254 Merck) and the products visualized with ultraviolet lamp (254 nm). ^1^H- and ^13^C-NMR spectra were determined in DMSO-*d*_6_ and CDCl_3_ solutions using a Varian spectrometer, operating at a frequency of 500.0 MH_z_ for proton and 125.70 MH_z_ for carbon. Peak positions are given in parts per million (d) from tetramethylsilane as internal standard, and coupling constant values (*J*) are given in Hz. Signal multiplicities are represented by: s (singlet), d (doublet), t (triplet), q (quadruplet) and m (multiplet). All described products showed ^1^H- and ^13^C-NMR spectra consistent with the assigned structures. Infrared (IR) spectra were obtained using spectrometer models 1420, 1600 FT-IR and Spectrum One FT-IR. Samples were examined as potassium bromide (KBr) disks. Melting points are uncorrected and were determined on a Fisatom (430-D) apparatus. All organic solutions were dried over anhydrous sodium sulfate and all organic solvents were removed under reduced pressure on a rotatory evaporator. 

#### 3.1.2. General Procedure for the Synthesis of 4-(1*H*-Pyrazol-1-yl)benzenesulfonamides **3a**–**g**

The functionalized amine **2** (0.4 mmol) was added to a solution of the appropriate arylsulfonyl chloride derivative (1.0 mmol) in THF (5 mL) containing triethylamine (0.5 mL). The reaction mixture was stirred for about 2 hours at room temperature, the end of the reaction was observed by TLC. The sulfonamide derivatives **3a**–**g** were isolated by addition of base and subsequent acidification. The products were recrystallized from ethanol, affording the sulfonamide derivatives in good yields [21].

*4-(4-Bromo-5-chloro-3-methyl-1H-pyrazol-1-yl)benzenesulfonamide* (**3a**). Brown powder, m.p. = 79–80 °C, Yield: 83%, IR (cm^−1^): 3051 (CH), 2928 (CH), 1599, 1527 and 1499 (C=C/C=N), 1334 and 1162 (SO_2_). ^1^H-NMR (DMSO-*d*_6_): 2.4 (s, 3H, CH_3_, pyrazole), 8.0 (s, 1H, NH), 6.3 (s, 1H, H4 pyrazole), 7.7 (d, 2H, *J* = 8.8 Hz, H2' and H6' phenyl), 7.3 (d, 2H, *J* = 8.8 Hz, H3' and H5' phenyl). ^13^C-NMR: 12.3 (CH_3_), 133.5 (C_3_ pyrazole), 10.2 (C_4_ pyrazole), 144.2 (C_5_ pyrazole), 140.7 (C_1'_ phenyl), 139.4 (C_4'_ phenyl), 121.4 (C_2'_ and C_6'_ phenyl), 130.9 (C_3'_ and C_5'_ phenyl). Anal. Calc. for C_10_H_9_N_3_ (%): C 34.26; H 2.59; N 11.98. Found (%) C 34.18; H 2.41; N 11.75. 

*4-(4-Bromo-5-chloro-3-methyl-1H-pyrazol-1-yl)-N-phenylbenzenesulfonamide* (**3b**). Brown powder, m.p. = 97-100 °C, Yield: 79%, IR (cm^−1^): 3051 (CH), 2928 (CH), 1599, 1527 and 1499 (C=C/C=N), 1334 and 1163 (SO_2_). ^1^H-NMR (DMSO-*d*_6_): 2.2 (s, 3H, CH_3_, pyrazole), 2.3 (s, 3H, CH_3,_ phenyl), 7.7 (d, 2H, *J* = 8.8, H2' and H6' phenyl), 7.4 (d, 2H, *J* = 8.8 Hz, H3' and H5' phenyl), 7.6 (d, 2H, *J* = 8.8 Hz, H2" and H6" phenyl), 7.2 (dd, 2H, *J* = 8.8 and 8.3 Hz, H3" and H5" phenyl), 6.2 (s, 1H, H4 pyrazole). ^13^C-NMR: 12.2 (CH_3_), 133.5 (C_3_ pyrazole), 100.2 (C_4_ pyrazole), 144.2 (C_5_ pyrazole), 140.4 (C_1'_ phenyl), 121.6 (C_2'_ and C_6'_ phenyl), 131.5 (C_3'_ and C_5'_), 138.5 (C_4'_ phenyl), 138.0 (C_1"_ phenyl), 121.5 (C_2"_ and C_6"_ phenyl), 130.5 (C_3"_ and C_5"_), 125.1 (C_4"_ phenyl). Anal. Calc. for C_16_H_13_N_3_ (%): C 45.03; H 3.07; N 9.85. Found (%) C 44.09; H 3.01; N 9.76. 

*4-(4-Bromo-5-chloro-3-methyl-1H-pyrazol-1-yl)-N-(4methylphenyl)benzenesulfonamide* (**3c**). Brown powder, m.p. = 158–160 °C, Yield: 78%, IR (cm^−1^): 3051 (CH), 2928 (CH), 1599, 1527 and 1499 (C=C/C=N), 1334 and 1162 (SO_2_). ^1^H-NMR (DMSO-*d*_6_): 2.4 (s, 3H, CH_3_), 7.6 (s, 1H, NH), 7.9 (dd, 2H, *J* = 8.8 Hz, H2' and H6' phenyl), 8.1 (dd, 2H, *J* = 8.8 Hz, H3' and H5' phenyl). ^13^C-NMR: 12.7 (CH_3_), 149.0 (C_3_ pyrazole), 96.3 (C_4_ pyrazole), 127.1 (C_5_ pyrazole), 143.9 (C_1'_ phenyl), 140.1 (C_4'_ phenyl), 124.8 (C_2'_ and C_6'_ phenyl), 127.1 (C_3'_ and C_5'_). Anal. Calc. for C_17_H_15_N_3_ (%): C 46.33; H 3.43; N 9.53. Found (%) C 46.27; H 3.34; N 9.41.

*4-(4-Bromo-5-chloro-3-methyl-1H-pyrazol-1-yl)-N-(4-bromophenyl)benzenesulfonamide* (**3d**). Brown powder, m.p. = 142–144 °C, Yield: 74%, IR (cm^−1^): 3051 (CH), 2928 (CH), 1599, 1527 and 1499 (C=C/C=N), 1334 and 1162 (SO_2_). ^1^H-NMR (DMSO-*d*_6_): 2.4 (s, 3H, CH_3_), 10.5 (s, 1H, NH), 7.9 (d, 2H, *J* = 8.3 Hz, H2' and H6' phenyl), 8.0 (d, 2H, *J* = 7.8 Hz, H3' and H5' phenyl), 7.2 (d, 2H, *J* = 7.8 Hz, H2" and H6" phenyl), 8.0 (dd, 2H, *J* = 7.8 and 8.3 Hz, H3" and H5" phenyl), 7.2 (t, 1H, 7.8 and 8.3, H4"). ^13^C-NMR: 12.8 (CH_3_), 149.2 (C_3_ pyrazole), 96.5 (C_4_ pyrazole), 126.7 (C_5_ pyrazole), 140.8 (C_1'_ phenyl), 124.7 (C_2'_ and C_6'_ phenyl), 129.3 (C_3'_ and C_5'_), 139.1 (C_4'_ phenyl), 137.4 (C_1"_ phenyl), 120.5 (C_2"_ and C_6"_ phenyl), 128.0 (C_3'_ and C_5'_), 124.5 (C_4'_ phenyl). Anal. Calc. for C_16_H_12_N_3_ (%): C 38.01; H 2.39; N 8.31. Found (%) C 37.49; H 2.23; N 8.29. 

*4-(4-Bromo-5-chloro-3-methyl-1H-pyrazol-1-yl)-N-(4-chlorophenyl)benzenesulfonamide* (**3e**). Brown powder, m.p. = −178–179 °C, Yield: 70%, IR (cm^−1^): 3051 (CH), 2928 (CH), 1599, 1527 and 1499 (C=C/C=N), 1334 and 1162 (SO_2_). ^1^H-NMR (DMSO-*d*_6_): 2.4 (s, 3H, CH_3_, pyrazole), 2.6 (s, 3H, CH_3_, phenyl) 10.7 (s, 1H, NH), 7.6 (d, 2H, *J* = 8.8 Hz, H2' and H6' phenyl), 8.0 (d, 2H, *J* = 8.8 Hz, H3' and H5' phenyl), 7.2 (d, 2H, *J* = 8.8 Hz, H2" and H6" phenyl), 7.9 (dd, 2H, *J* = 8.8 and 8.3 Hz, H3" and H5" phenyl). ^13^C-NMR: 12.7 or 12.6 (CH_3_ pyrazole or phenyl), 149.2 (C_3_ pyrazole), 96.8 (C_4_ pyrazole), 136.9 (C_5_ pyrazole), 148.2 (C_1'_ phenyl), 132.2 (C_2'_ and C_6’_ phenyl), 132.5 (C_3'_ and C_5'_). 140.9 (C_4'_ phenyl), 138.7 (C_1"_ phenyl), 128.0 (C_2"_ and C_6"_ phenyl), 137.9.0 (C_3'_ and C_5'_). 124.7 (C_4'_ phenyl). Anal. Calc. for C_16_H_12_N_3_ (%): C 41.67; H 2.62; N 9.11. Found (%) C 41.55; H 2.57; N 9.03. 

*N-[4-(5-Chloro-3-methyl-1H-pyrazol-1-yl)phenyl]benzenesulfonamide* (**3f**). Brown powder, m.p. = 169–170 °C, Yield: 70%, IR (cm^−1^): 3051 (CH), 2928 (CH), 1599, 1527 and 1499 (C=C/C=N), 1334 and 1162 (SO_2_). ^1^H-NMR (DMSO-*d*_6_): 2.4 (s, 3H, CH_3_, pyrazole), 7.7 (s, 1H, H_4_ pyrazole), 10.7 (s, 1H, NH), 7.4 (d, 2H, *J* = 8.8 Hz, H2' and H6' phenyl), 8.0 (d, 2H, *J* = 8.8 Hz, H3' and H5' phenyl), 7.2 (d, 2H, *J* = 8.8 Hz, H2" and H6" phenyl), 7.9 (dd, 2H, *J* = 8.8 and 8.3 Hz, H3" and H5" phenyl). ^13^C-NMR (d, ppm):12.6 (CH_3_ pyrazole), 149.0 (C_3_ pyrazole), 96.6 (C_4_ pyrazole), 128.6 (C_5_ pyrazole), 140.8 (C_1'_ phenyl), 124.4 (C_2'_ and C_6'_ phenyl), 127.9 (C_3'_ and C_5'_). 138.7.9 (C_4'_ phenyl), 136.3 (C_1"_ phenyl), 121.9 (C_2"_ and C_6"_ phenyl), 129.0 (C_3'_ and C_5'_). 124.9 (C_4'_ phenyl). Anal. Calc. for C_16_H_13_N_3_ (%): C 45.03; H 3.07; N 9.85. Found (%) C 49.93; H 2.94; N 9.77. 

*N-[4-(5-Chloro-3-methyl-1H-pyrazol-1-yl)phenyl]-4-methylbenzenesulfonamide* (**3g**). Brown powder, m.p. = 215–217 °C, Yield: 73%, IR (cm^−1^): 3051 (CH), 2928 (CH), 1599, 1527 and 1499 (C=C/C=N), 1334 and 1162 (SO_2_). ^1^H-NMR (DMSO-*d*_6_): 2.3 (s, 3H, CH_3_ pyrazole), 7.7 (s, 1H, H_4_ pyrazole), 7.6 (d, 2H, *J* = 8.8, H2' and H6' phenyl), 7.8 (d, 2H, *J* = 8.8 Hz, H3' and H5' phenyl), 6.9 (d, 2H, *J* = 8.8 Hz, H2" and H6" phenyl), 7.1 (dd, 2H, *J* = 8.8 and 8.3 Hz, H3" and H5" phenyl). ^13^C-NMR: 13.6 or 12.7 (CH_3_ pyrazol or phenyl), 126.1 (C_3_ pyrazole), 168.9 (C_4_ pyrazole), 148.5 (C_5_ pyrazole), 134.2 (C_1'_ phenyl), 120.8 (C_2'_ and C_6'_ phenyl), 120.9 (C_3'_ and C_5'_), 129.1 (C_4'_ phenyl), 137.6 (C_1"_ phenyl), 127.9 (C_2"_ and C_6"_ phenyl), 129.6 (C_3'_ and C_5'_). 143.3(C_4"_ phenyl). Anal. Calc. for C_17_H_15_N_3_ (%): C 46.33; H 3.43; N 9.53. Found (%) C 46.17; H 3.33; N 9.41. 

### 3.2. Pharmacology

#### 3.2.1. *In Vitro* AntiLeishmanial Drug Assay

Parasites in metacyclic phase (4 × 10^6^ parasites/mL) were incubated with the derivatives (40–160 mg/mL) solubilized in dimethyl sulphoxide (DMSO, Sigma Chemical Co., St. Louis, MO, USA) at 26 °C for 24 h [20]. The results were expressed as IC_50_/24 h, the concentration of a compound that caused a 50% reduction in survival/viability compared with non-treated culture. Pentamidine was used as reference drug and all tests were carried out in triplicate. Promastigotes of *L. amazonensis* (MHOM/BR/77/LTB0016 strain) and *L. infantum (syn. chagasi)* (MCAN/BR/97/P142 strain) were grown in Schneider’s insect medium (Sigma) at 26 °C in pH 7.2 supplemented with 10–20% (v/v) heat-inactivated fetal calf serum.

#### 3.2.2. *Animals*

The BALB/c mice from Laboratory Animals Nucleus (UFF) were sacrificed to obtain peritoneal cells and for both infection and isolation of *Leishmania*. The protocol assays was approved by the Institutional Committee of the Center for Biological Evaluation and Care of Research Animals (CEUA-UFF).

#### 3.2.3. *In Vitro* Cytotoxicity

BALB/c mice peritoneal cavity cells (4 × 10^5^cells/well) were incubated with the derivatives (10, 20, 40 and 80 µg/mL) in 96 wells plate for 24 h in cold RPMI 1640 medium, supplemented with 1 mmol·L^−1^L-glutamine, 1 mol·L^−1^ HEPES, penicillin G (10^5^ IU·L^−1^) and streptomycin sulfate (0.10 g·L^−1^) at 37 °C in a humidified 5% CO_2_ atmosphere. After that, 3-[4,5-dimethylthiazol-2-yl]-2,5-diphenyltetrazolium bromide, MTT (Sigma) was added and the reaction was interrupted with DMSO after 2 h. The results were determined in 540 nm by using a MicroQuant spectrophotometer (Biotek-Instrument Inc., Winooski, VT, USA). All assays were repeated at least four times in quadruplicate. The cytotoxicity profile was expressed as CC_50_/24 h, the concentration of a compound that caused cytotoxicity compared to non-treated cultures [6]. Index of selectivity (IS) was defined as the ratio of the CC_50_ value on the macrophage to the IC_50_ value on the *L. amazonensis* or *L. infantum* strains (promastigotes). 

#### 3.2.4. Molecular Modeling Studies:

All molecular computations were performed using SPARTAN’08 (Wavefunction Inc. Irvine, CA, USA) as described elsewhere [20]. The theoretical studies of druglikeness and drugscore and ADMET were performed using Osiris Property Explorer (http://www.organic-chemistry.org/). Briefly the structures were optimized to a local minimum and the equilibrium geometry obtained in vacuum using RM1 semi-empirical methods. Subsequently, molecules were submitted to a single-point energy *ab initio* calculation, at the 6-31G* level, to calculate some stereoelectronic properties and perform the SAR studies. Thus, we calculated for all compounds best conformation the values of HOMO (Highest Occupied Molecular Orbital) and LUMO (Lowest Unoccupied Molecular Orbital) energies and density isosurface, molecular weight (MW), molecular surface area and volume, polar surface area (TPSA), dipole moment and lipophilicity using the same program. 

The druglikeness value is calculated based on the occurrence frequency of each fragment is determined within the collection created by shredering 3300 traded drugs as well as 15,000 commercially available chemicals (Fluka Chemical Co., Buchs, Switzerland) yielding a complete list of all available fragments. In this case, positive values point out that the molecule contains predominantly the better fragments, wich are frequently present in commercial drugs but not in the non-druglike collection of fluka compounds. The drugscore combines druglikeness, clogP, logs, molecular weight and toxicity risks in one handy value that may be used to judge the drug potential of a compound. 

#### 3.2.5. Statistical Analysis

Each experiment was done three to four times, in triplicate. Significance was determined using a non-paired *t* Student test and Mann–Whitney analyses and *p* < 0.05. 

## 4. Conclusions

In summary, a novel family of 4-(1*H*-pyrazol-1-yl)benzenesulfonamide derivatives **3a**–**g** has been synthesized and evaluated against *Leishmania spp.* The antileishmanial data showed a better active profile for **3b**–**e** on promastigote forms of *L. infantum* and *L. amazonensis*, close to that of the reference drug pentamidine. The cytotoxic tests using murine adherent peritoneal cells pointed out that **3b** and **3e** had better IC_50_ values than pentamidine. Molecular modeling evaluation indicated that changes in electronic regions, orientation as well as lipophilicity of the derivatives were areas to improve the interaction with the parasitic target. The biological and theoretical data reinforced the potential of these molecules as an alternative option for testing against resistant *Leishmania* strains and/or for further synthetic and biological exploration for the development of better antileishmanial drugs.
